# An allele separating skeletal patterning and spermatogonial renewal functions of PLZF

**DOI:** 10.1186/1471-213X-10-33

**Published:** 2010-03-25

**Authors:** Yung-Hao Ching, Lawriston A Wilson, John C Schimenti

**Affiliations:** 1 Dept. of Biomedical Sciences, Cornell University, Ithaca, NY 14850 USA; 2 The Jackson Laboratory, Bar Harbor, ME 04609 USA; 3 Current address: National Laboratory Animal Center. PO Box 1-86, Nankang, Taipei, 115 Taiwan

## Abstract

**Background:**

The promyelocytic leukemia zinc finger gene *Plzf *(also called *Zbtb16, Zfp145 *or *Green's luxoid*) belongs to the POZ/zinc-finger family of transcription factors. It contains a BTB/POZ domain that mediates epigenetic transcriptional repression. PLZF is essential for proper skeleton patterning and male germ cell renewal. Two alleles have been reported that display similar phenotypes: a targeted knock-out, and the spontaneous nonsense mutation *luxoid*.

**Results:**

We describe a new ENU induced missense allele of *Plzf *called seven toes (*Plzf*^7*t*^). Homozygous animals exhibit hindlimb and axial skeleton abnormalities. Whereas the skeletal abnormalities are similar to those of the other alleles, *Plzf*^7*t *^differs in that it does not cause spermatogonial depletion and infertility. Positional cloning revealed a point mutation changing the evolutionarily conserved amino acid Glu44 to Gly, possibly altering the BTB domain's activity.

**Conclusions:**

*Plzf*^7*t *^is a separation-of-function allele that reveals differential requirements for domains of PLZF in different developmental milieus.

## Background

*Plzf *is a widely studied gene due to its involvement in varied biological processes. It was originally discovered as being fused with the *RARA *(retinoic acid receptor alpha) gene in translocations underlying acute promyelocytic leukemias in humans [1]. The most prominent phenotypic features of *Plzf *null mice are defects in patterning of limb and axial skeleton structures [2]. Affected animals exhibit homeotic-like anterior-to-posterior transformations in the axial and appendicular skeleton, causing hindlimb abnormalities such as extra digits and conversion of the fibula to a tibia-like structure. The spontaneous mutation luxoid (*lu*), later shown to be an allele of *Plzf*, is characterized by similar, albeit semidominant, limb abnormalities and recessive skeletal defects [3-5].

A critical function of PLZF is in spermatogonial stem cell renewal. Both the null and luxoid alleles cause progressive male infertility due to depletion of the spermatogonial stem cell pool with age [3,6]. In the absence of PLZF, the balance between self-renewal and differentiation of spermatogonial stem cells is disrupted. PLZF is present as nuclear foci in Type A-single (putative stem cells), A-paired (undifferentiated spermatogonia), and A-aligned (differentiating) spermatogonia, but not the more differentiated Type B spermatogonia or in subsequent meiotic spermatocytes or postmeiotic spermatids [3].

PLZF contains nine Krüppel-type sequence-specific zinc finger DNA binding domains and a BTB/POZ protein-protein interaction domain in its N terminus. The latter allows BTB domain-containing proteins to form homo- or hetero-dimers [7,8]. BTB-containing proteins, including PLZF, function as transcriptional repressors that affect gene expression via chromatin remodeling [7,9,10]. The repressive activity of the BTB domain can be autonomous, or it can mediate interactions with co-repressors that in turn recruit histone deacetylases, resulting in epigenetic silencing [11].

The skeletal patterning defects of *Plzf *null mice (officially *Zbtb16*^*tm*1*Ppp*^; abbreviated *Plzf*^-/-^) have been linked to failed transcriptional repression of the *HoxD *complex genes [2,12]. PLZF was found to bind promoter regions in the AbdB* HoxD* complex, directly interacting with polycomb proteins, and thereby recruiting histone deacetylases to silence gene expression [12]. The spermatogonial stem cell renewal defects in *Plzf *mutants have been linked to failed transcriptional silencing of *Kit *[13]. The KIT receptor is present in differentiating spermatogonia in adult mice, and is required for the first wave of prepubertal spermatogonial expansion [14]. Therefore, KIT is a critical factor in spermatogonial differentiation, and the control of its expression affects the balance of stem cell maintenance. PLZF directly binds the *Kit *promoter, and PLZF-deficient mice have elevated *Kit *transcription [13], presumably resulting in a tipping of the balance from stem cell maintenance towards differentiation, and thus eventual exhaustion of the spermatogonial stem cell pool.

Here, we report a separation-of-function allele of *Plzf *called *seven toes *(*Plzf*^7*t*^; officially *Zbtb16*^7*t*^), which was recovered in an ENU mutagenesis screen [15]. This allele exhibits skeletal phenotypes similar, but not identical, to the luxoid and null *Plzf *alleles. Importantly, *Plzf*^7*t *^lacks the germ cell depletion phenotypes of all previously reported *Plzf *alleles. *Plzf*^7*t *^has a missense mutation in the BTB domain, which must disrupt a molecular activity involved in normal skeleton morphogenesis that is separate from that required for silencing of *Kit *in spermatogonial regeneration.

## Results

### Generation and positional cloning of the Seven toes (*7t*) mutation

*7t *arose in an N-ethyl-N-nitrosourea (ENU) mutagenesis screen that employed a balancer chromosome to detect mutations on proximal mouse chromosome 5 [15]. Affected animals exhibited recessive limb patterning defects (the most overt being one or two extra digits on one or both hindlimbs; described below in greater detail) without other obvious abnormalities. F1 intercrosses indicated Mendelian segregation of the limb phenotype. However, it was evident that *7t *was unlinked to Chromosome 5.

**Figure 1:**
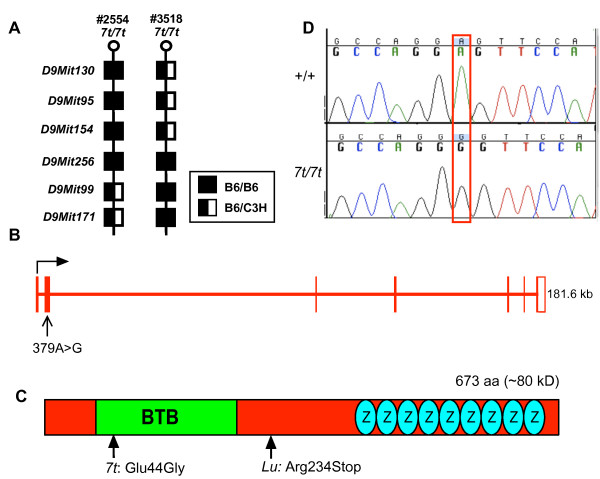
**Positional cloning of the *seven toes *(*7t*) mutation**. (A) Shown are the genotyping results for two affected animals (2554 and 3518, both showing polydactyly) harboring critical recombination events. This localized *7t *to a 0.65 Mb interval between SSLP markers *D9Mit154 *and *D9Mit99*. (B) The intron/exon structure of *Plzf *on mouse chromosome 9. (C) Sequencing chromatograms of wild type (*+/+*) and *Plzf*^7*t*/7*t *^cDNA reveal an A to G change at nt 379 of the *Plzf *cDNA. (D) Schematic diagram of the PLZF proteins with the indicated BTB and zinc-finger (ovals with "Z") domains. The *7t *mutation changes codon 44 from glutamic acid to glycine.

**Figure 2:**
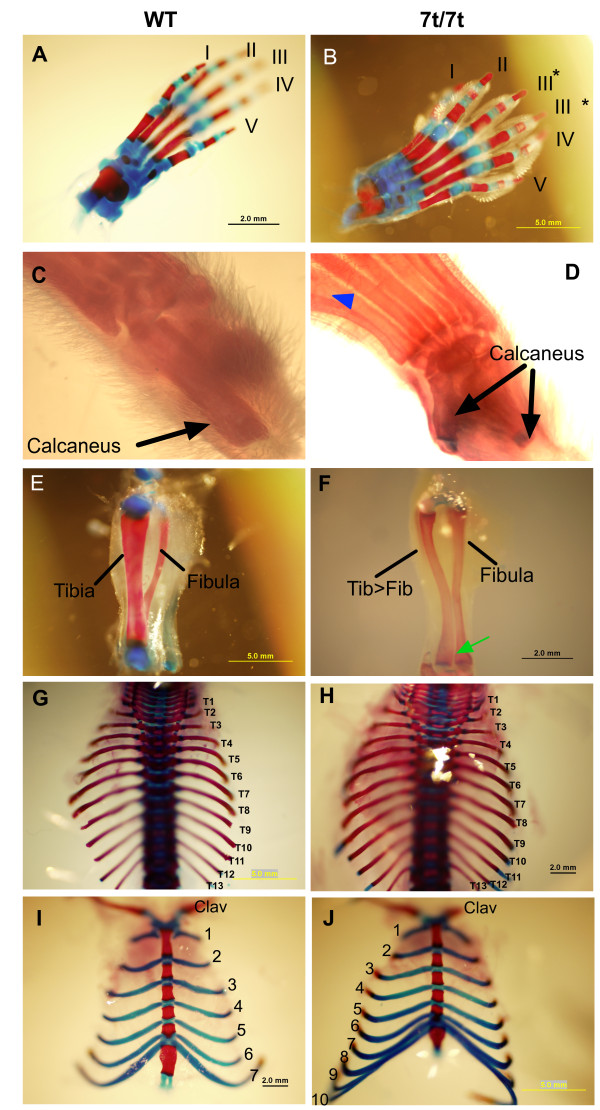
**Limb and axial skeleton phenotypes of the *7t *mutant**. Panels on the left are wild type, and panels on the right are mutants. (A) Digits I-V are anterior to posterior, respectively. (B) Representative example of a *Plzf*^7*t*/7*t *^hindlimb in which digit III has been duplicated (asterisk). (C) Wild-type has one calcaneus. (D) The tatus of the *Plzf*^7*t*/7*t *^is homeotically transformed into a calcaneus. The blue triangle indicates a metatarsal fusion of digits I and II. In contrast to the wild-type hindlimbs (E), the mutants (F) display a thinner tibia that appears to be homeotically transformed into a thicker fibula-like structure. The green arrow indicates a failure of the two bones to fuse. (G, H) A dorsal view shows that the total number of thoracic vertebrae are the same in both genotypes. However, whereas the wild type has seven ribs attached to the sternum (I), *Plzf^7t/7t^*mutants have up to 10 (J). "Clav" = clavicle.

To positionally clone *7t*, an F2 mapping cross was conducted, resulting in its localization to a 0.65 Mb interval on Chr 9 between *D9Mit154 *and *D9Mit99 *(Fig. 1a). According to the July 2007 mouse genome assembly (UCSC Genome Browser), the critical region contains 8 annotated RefSeq genes (see Additional file 1). The coding regions of 5 were PCR-amplified from genomic DNA and/or cDNA, and were scanned by dHPLC to identify DNA sequence alterations. A variation was detected in *Plzf*, which consists of 7 exons spanning 181.6 kb of genomic DNA (Fig. 1b). *Plzf *is annotated to produce a 5,114 nt transcript encoding a 673 amino acid protein (Fig. 1c). As described earlier, mutations in *Plzf *cause skeletal phenotypes similar to those of *7t *(Fig. 2; described later). The amplimer of *Plzf *that contained a dHPLC variant was sequenced, revealing an A to G transition at nucleotide 379 of the *Plzf *cDNA (NM_001033324; Fig. 1d). This is predicted to alter amino acid 44 from the polar and acidic amino acid glutamic acid, into the nonpolar neutral amino acid glycine (Glu44Gly). This amino acid resides in the conserved BTB domain of PLZF (Fig. 1c).

### *7t *is allelic to *Plzf*^*lu*^

**Figure 3:**
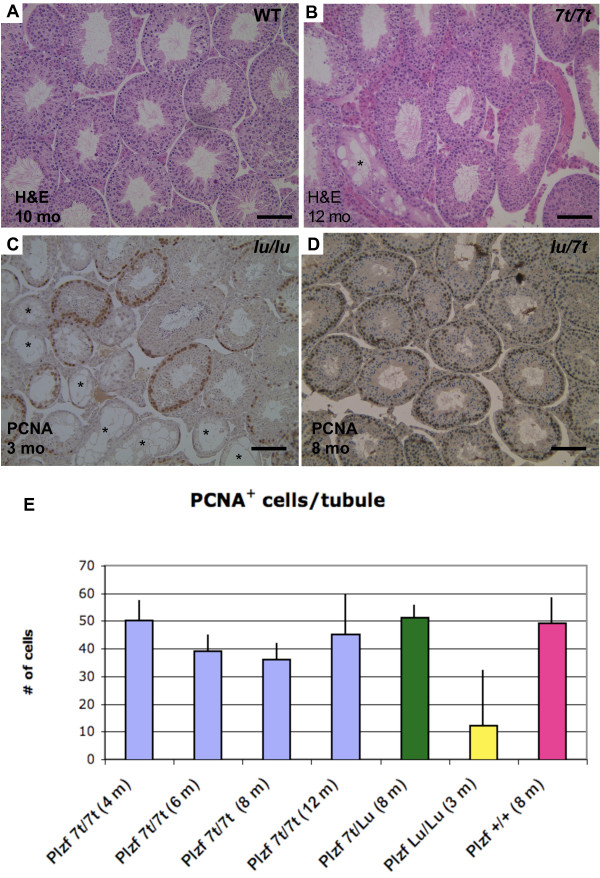
**Testis histopathology and germ cell quantification**. (A, B) H&E staining wildtype and *7t*/*7t *testis sections. (C, D) PCNA immunolabeling of *lu/lu *and *lu/7t *testis sections. PCNA is a marker of G1 and S phase spermatogonia, which are present near the periphery of the tubules (dark brown staining). *Plzf*^7*t*/*lu *^(D) and +/+ or *Plzf*^7*t*/7*t *^testes (not shown) show no sign of progressive germ cell loss after several months of age, in contrast to luxoid testes (C) which show dramatic loss even at 3 months of age. The size bars are 0.1 mm. Asterisks indicate germ cell-depleted seminiferous tubules. (E) Percentage of PCNA positive cells per seminiferous tubule section. Genotypes and ages of the mice are indicated below the graph. *Plzf*^7*t*/7*t *^testes had an average of 50 ± 7.5, 39 ± 6.18, 36 ± 6.22 and 45 ± 14.84 PCNA+ cells/tubule at 4, 6, 8 and 12 months of age, respectively, similar to 8 month old wildtype (49 ± 9.54) and *Plzf*^7*t*/*lu *^(51 ± 4.7). Three month old *Plzf*^*lu*/*lu *^testes had 12 ± 20.4.

To determine if the Glu44Gly mutation in *Plzf *is responsible for the *7t *phenotype, we performed complementation tests with the *Plzf*^*lu *^(*lu*) allele. In crosses of *7t/7t *to *lu/+ *mice, a total of 71 pups were born, of which 33 displayed polydactyly of the hindlimb and anterior-posterior transformation in the axial skeletal (detailed below); DNA sequencing revealed that all 33 were *7t/lu*. The failure to complement demonstrates that *7t *is an allele of *Plzf*, which we will refer to as *Plzf*^7*t *^(or *7t*). However, like *7t *homozygotes, *lu/7t *males had testes of normal size, and there was no indication of substantial germ cell depletion in testis histology (Fig. 3a, b). Therefore, *7t *appears to be a separation-of-function mutation in which skeletal patterning is affected but not germ cell maintenance. We also noticed that the *7t *allele rescued the growth retardation and pre-wean lethality of the *lu/lu *animals. A summary of the similarities and differences between *7t *and other *Plzf *alleles, incorporating data presented below, is presented in Table 1. Notably, *lu *is semidominant in that heterozygotes exhibit skeletal defects unlike either *7t *or the null allele [2,4]. It is possible that *lu *is more severe due to dominant negative effects of a truncated protein that it potentially encodes (see Fig. 1c).

**Table 1:** Complementation testing and comparative phenotypes of *luxoid *and *7t *mice

	*Plzf*^*7t/7t*^	*Plzf*^*lu/lu*^	*Plzf*^*lu/7t*^
Genetic background	C3H	B6	Mixed (C3H/B6)
Skeletal phenotypes	Yes	Yes	Yes
Male Sterility	No	Yes	No
Testis Size	Normal	Small	Normal
Female Fertility	Normal	Sub-fertile	Normal
Pre-Wean Lethality	No	Yes	No
Growth Retardation	No	Yes	No

### *Plzf*^*7t *^causes skeletal abnormalities

Mouse limbs contain three basic segments. Proximal to distal, these are: (1) the stylopod (humerus/femur); (2) the zeugopod, containing two skeletal elements (radius-ulna and tibia-fibula); and (3) the autopod, comprised of the wrist/ankle and digits. *Plzf*^7*t*/7*t *^mice exhibit defects in all three segments of the hindlimb (Figs. 2b, d, f), but not the forelimb. It was reported that 5% of *Plzf*^-/- ^mice had forelimb defects in addition to the hindlimb abnormalities [2]. The polydactyly phenotype in the autopod of *7t/7t *(between N4 and N5) hindlimbs was 100% penetrant (Figs. 2b); all 302 genotype-verified homozygotes had polydactyly, compared to 92% reported for null mutants [2]. However, other hindlimb structures were not as severely affected by the *7t *allele compared to the knockout (see Table 2), including the metatarsals. In 20% of *7t/7t *limbs, the talus (which is proximal to the first three digits, forming lower part of the ankle joint in humans) was transformed into a calcaneus (heel; Fig. 2d). 20% of the hindpaws had webbing between digits I and II (Fig. 2d), and we observed autopod metatarsal fusions in 13% of mice (Fig. 2d).

**Table 2:** Comparison of *Plzf*^-/- ^and *Plzf*^7*t*/7*t *^skeletal phenotypes

Structure	Defect	*Plzf*^-/-^	*Plzf*^7*t*/7*t*^
Hindlimb	digit I>II transformation	92%	100%
	preaxial digit resembles digit V or digit I absence	40%	20%
	Talus>calcaneus transformation	90%	53%
	interdigital webbing	60%	20%
	anterior C1 to navicular	38%	
	C1 to cuboid bone	42%	
	metatarsal fusion	15%	13%
	metatarsal bony outgrowths	69%	40%
	absent tibia	98%	
	separation of tibia and fibula	96%	47%
	thickened fibula	32%	40%
	tibia to fibula transformation	38%	40%
	shortened zeugopod/absent fibula	16%	33%
	duplication or absence of patella	52%	0%
	absence of meniscus	28%	0%
Forelimb	preaxial polydactyly	5%	0%
	interdigital webbing	5%	0%
Axial skeletal	14 thoracic vertebrae	100%	0%
	extra pair of ribs attached to sternum		73%
	malformed xiphoid process	100%	40%
	T10 to T11 vertebrae shift	70%	n/a
	homeotic transformation of sacral region	80%	53%
	Shortening and kinked tail	100%	0%

In the zeugopod region, the tibia and fibula failed to fuse in 47% of *7t/7t *mice (*vs*. 96% of null animals; Fig. 2f). Additionally, 40% of fibulae were thickened and 40% of tibiae were transformed to fibulae (compared to 32% and 38%, respectively, in *Plzf*^-/-^; Fig. 2f). There was a shortening of zeugopod/absence of fibula in 33% of limbs, but no animals develop duplication or absence of the patella or meniscus (data not shown).

All of the *7t/7t *animals (N4-N5 backcross generations into strain C3HeB/FeJ) had 13 thoracic vertebrae as in wild-type, (Figs. 2g, h), but 70% of *Plzf*^-/- ^mice were reported to have 14 [2]. The additional segment of *Plzf*^-/- ^vertebrae is associated with an extra pair of ribs that also attached to the sternum. Unlike the wild type, 73% of *7t/7t *mice had elongated 8^th ^and 9^th ^ribs that attached to the sternum (Fig. 2i, j). All *Plzf*^-/- ^mice had malformed xiphoid processes (lower part of sternum) compared to only 40% of the *7t/7t *mice (data not shown). Unlike the nulls, none of the *7t *homozygotes had a truncated and kinked tail. These results demonstrated that *7t *causes a similar but milder axial phenotype than the reported knock-out allele, but the polydactyly phenotypes are essentially identical with higher penetrance. Table 2 summarizes the skeletal phenotypes of null and *7t/7t *mice.

### *7t/7t *mice do not exhibit spermatogonial depletion

Whereas *lu/lu *and *Plzf*^-/- ^males undergo male germ cell depletion and infertility due to defective spermatogonial stem cell renewal, *7t *homozygotes were normally fertile. In matings to C3HeB/Fej (C3H) females, C3H semi-congenic males (N4-N5) sired an average of 6.5 ± 1.9 pups compared to 5.6 ± 2.3 for C3H control males (p = 0.036), indicating the *7t *does not impact fertility negatively. Notably, *lu/lu *males co-isogenic on C3H/He (the strain on which *lu *arose) were found to be infertile (Green, 1955), thus making it unlikely that genetic background is responsible for the disparate *lu *and *7t *fertility phenotypes.

*lu/lu *and *Plzf*^-/- ^adults have small testes and markedly decreased numbers of germ cells in seminiferous tubules. This Sertoli Cell Only-like syndrome becomes more prominent with age as the stem cell pool declines [3,6]. *7t/7t *males did not exhibit small testes at any age. Nevertheless, we performed histological analysis of their testes to reveal potential germ cell depletion. There were no agametic tubules in young mice, unlike the null and *luxoid *alleles (Fig. 3a). Only at older ages (> 10 months) did some mice exhibit Sertoli Cell only tubules (< 2% of tubules; Fig. 3b) compared to <1% in wildtype (Fig. 3a). To quantify any possible minor effects on the germ cell pool, testis immunostaining for proliferating cell nuclear antigen (PCNA), a marker of cells in early G1 and S phases of the cell cycle, was performed on *7t/7t *mice at 4, 6, 8 and 12 months, *lu/lu *mice at 3 months, and wild types at 8 months of age. Twenty randomly selected tubules from each testis were counted for PCNA^+ ^cells. PCNA^+ ^cells remained present in most tubules of *7t/7t *testes at 8 months (data not shown) of age and older, with no evidence of quantitative decline (Fig. 3e). This contrasts with the *lu/lu *and null mutants, where over 50% of tubules were PCNA-negative at 8 months of age (Fig. 3c) [3,6]. The *7t *allele also rescued the male germ cell depletion phenotype of *lu*, as *lu/7t *had normal testes (Fig. 3d). These data indicate that the *7t *allele does not appreciably affect spermatogonial stem cell maintenance.

### Expression of PLZF and predicted effect of the *7t *mutation on PLZF's BTB domain

**Figure 4:**
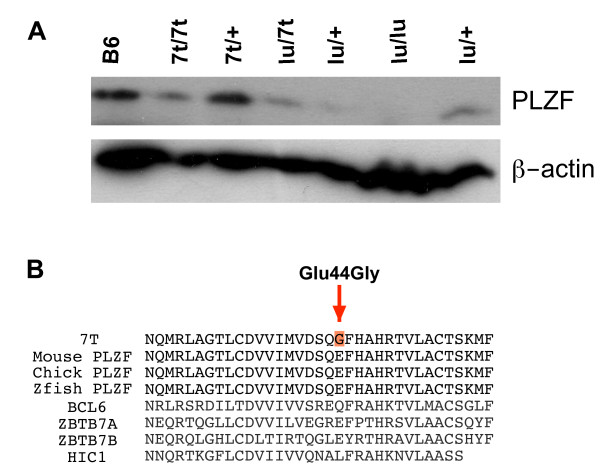
**Expression of PLZF and alignment of BTB domains in region of *7t *mutation**. (A) Western blot analysis of testis proteins from the indicated genotypes with the mouse anti-PLZF antibody. All the protein samples were prepared from testes of adult animals (>3 months) except for the *lu/7t *and *lu/lu *samples, which were from 1 month old mice; this was to ensure that the result was not falsely negative due to depletion of spermatogonia (the cells producing PLZF) that occurs over time into adulthood in *lu *mutants. (B) The *7t *mutation region is highly conserved amongst vertebrate *Plzf *alleles, but not other BTB domains in BTB domain-containing zinc finger proteins. Zfish = *Danio rerio*; chick = *Gallus gallus*.

To assess the stability of PLZF^7*t*^, Western blot analysis of testis protein from various control and mutant genotypes was performed. A species of the predicted full-length size of PLZF (~80 kDa) was present in WT, *7t/7t*, *7t*/+ and *7t/lu *but not *lu/lu *(Fig. 4a), which contains a nonsense mutation in codon 254 of the 673 amino acid protein (Fig. 1c) and thus should serve as negative control. These data indicate that the *7t *mutation does not decrease, to any major qualitative degree, the level of PLZF in mutant testes relative to various control genotypes (compare PLZF signals to β-actin loading reference). Thus, we surmise that the amino acid change encoded by the *7t *mutation alters PLZF function.

Alignment of mouse PLZF amino acid sequence surrounding residue 44 indicated that this region is highly conserved amongst vertebrates including zebrafish (*Danio rerio*) and chicken (*Gallus gallus*) (Fig. 4b). However, the particular mutated amino acid (GLU44 in WT mouse PLZF) is not absolutely conserved in the BTB domains of all proteins of the BTB-ZF (BTB domain-containing zinc finger proteins) family (Fig. 4b).

The BTB domain structure of human PLZF was determined at 1.9A resolution, revealing a tightly intertwined dimer [16]. Amino acid residue 44 is located in the 3^rd ^β-sheet of the BTB domain, in a charged pocket that facilitates dimerization [16]. It has been shown that mutations that cause misfolding of the BTB domain result in failed dimerization and therefore nonfunctional protein products [11]. The proper charge alignment in the BTB pocket is required for PLZF transcriptional repression via histone deacetylase (HDAC) recruitment [11]. Therefore, the Glu44Gly mutation may alter both the conformation and the charge of the BTB domain, thus affecting its function.

## Discussion

Through a forward genetics approach, we isolated a new allele (*7t*) of *Plzf*. The *7t/7t *mice displayed skeleton abnormalities similar to the null and nonsense (*lu*) alleles, without the male germ cell depletion phenotype observed in the two previously reported alleles. A Glu44Gly mutation was found in the evolutionarily conserved BTB domain, which mediates protein-protein interactions. The Glu44Gly may weaken, disrupt or change the conformation of interactions between PLZF and itself or other BTB/POZ domain-containing proteins, thereby affecting the expression of genes involved in skeletal patterning. The expression of *Hoxd *genes, *Hoxa13 *and bone morphogenetic proteins (BMPs) are misregulated in *Plzf*^-/- ^mice, however, expression of *Shh *and *Fgf4 *is not altered [2]. Therefore, *Plzf *is likely to mediate aspects of anterior-posterior pattering in both the axial and limb skeleton through the BTB/POZ domain.

The lack of a germ cell depletion phenotype indicates that *7t *is a separation-of-function mutation that can provide insight into the functional domains of PLZF. Given existing information, there are two likely mechanistic explanations for why the mutation specifically affects skeletal patterning. One is that alteration of the BTB domain abolishes co-repressor(s) binding in cell types involved in skeletal development, but does not alter binding of co-repressor(s) in germ cells required for silencing *Kit*. A second possibility is that the BTB domain is not required for PLZF's role in repressing *Kit *expression in spermatogonia. We hypothesize that since the zinc finger domains remain intact in the *7t *allele, that sequence-specific binding of PLZF^7*t *^to the *Kit *promoter is unaffected by the BTB domain disruption. Interestingly, a human patient with skeletal defects and genital hypoplasia was identified as carrying a missense allele of *PLZF *in *trans *to a deletion [17]. The nonconservative Met617Val alteration occurred in a zinc finger motif, causing functional disruption in reporter assays. These data suggest that the Zn finger domains (and presumably, DNA binding) are important for both skeletal and germ cell functions. This patient also had phenotypes that were not apparent or tested in any of the mouse *Plzf *mutant models, including severe mental retardation, short stature, dystrophy, microcephaly, cranial dysmorphisms, and cryptorchidism. It is likely that many of these phenotypes are related to the deletion, rather than homozygous loss of PLZF, since other patients with deletions in this region of 11q share the non-genital phenotypes [17].

The *7t/7t *mutation was induced on strain C57BL/6J (B6) and backcrossed into C3H for five generations (N5). In the earlier generations (N1 to N3), the hindlimbs displayed more severe abnormalities (especially in the zeugopod region) than in later generations (N4 & N5). This may explain why the limb and axial skeleton phenotypes of *7t/7t *mice were not as severe as in null mice (studied in a 129xB6 mixed background). During the positional cloning process, *Plzf*^7*t *^was outcrossed to several different inbred strains, including CAST/Ei. More than 147 F2 offspring from the CAST/Ei crosses were recovered, yielding the expected number of F2 homozygotes, but none of these displayed polydactyly. This presumably dominant modifier effect was not seen in similar crosses to strains BALB/cJ or DBA/2J. A similar modifier effect was observed in *Twist *mutants [18] and *Strong's luxoid *[19]. These observations open the door to the potential identification of modifiers that function in the same developmental pathway as PLZF. Homozygous *7t *males were fertile on all strain backgrounds; thus the phenotype modifications appear limited to skeletal defects.

## Conclusions

This work demonstrates the utility of forward genetic approaches for the dissection of functional domains within a protein, and the roles of those domains in various cell types and developmental processes. The *7t *separation of function mutation will be informative for eventual mechanistic elucidation of how this protein acts in different milieus, namely, skeletal development and in the germ lineage.

## Methods

### Animals

The mice used in this study were maintained first at The Jackson Laboratory, and later at Cornell University. The use of mice for these experiments was approved by the Institutional Animal Care and Use Committees (IACUC) of both institutions.

### Skeletal analysis

Skin and internal viscera were removed from mice, fixed overnight in 100% ethanol, followed by 4 days in acetone. The specimens were stained with Alcian blue and Alizarin red for 10 days followed by overnight treatment with 2% KOH. Samples were stored in a solution of 40% glycerol, 40% ethanol and 20% benzoalcohol.

### Linkage mapping of *Plzf*^*7t*^

*Plzf*^7*t *^arose on the strain C57BL/6J. Homozygous mutants were crossed to C3HeB/FeJ to generate F1 heterozygotes, which were intercrossed to yield 411 F2 progeny. Affected offspring were PCR genotyped with a panel of polymorphic microsatellite markers distributed across the genome, and association of the phenotype with homozygosity for B6 alleles at microsatellite loci revealed linkage.

### Mutational Analysis using heteroduplex analysis

Denaturing HPLC analysis using a Transgenomic WAVE machine was employed to scan for mutations in candidate genes within the *7t *critical region. PCR primers were designed to amplify either cDNA or genomic sequences from heterozygous experimental and control animals. If sequence polymorphisms were detected in the dHPLC analysis, those amplicons were sequenced to identify the exact nucleotide change.

### Histology and immunohistochemistry

Testes were fixed in Bouin's, embedded in paraffin, sectioned, and stained with hematoxylin and eosin (H&E). For immunohistochemistry, the sections were deparaffinized and dehydrated through Xylene/Ethanol/PBS gradients. Slides were subjected to antigen retrieval by boiling at 95°C for 40 minutes in 10 mM Tris-sodium citrate/PBS (pH = 6.0) with 0.05% Tween 20, followed by quenching and blocking in 3% H_2_O_2_/methanol for 15 min. A M.O.M. kit (Vector Laboratories) was used for blocking according to the manufacturer's instructions. The sections were incubated with a mouse monoclonal anti-PCNA antibody (clone PC10; Sigma; diluted 1:200) at 4°C overnight. Histostain-SP Kit (AEC, Broad Spectrum, Zymed^®^) was used to visualize binding.

### Sperm counts

Epididymides were minced with small scissors in 1 ml PBS then incubated at 37° for 15 minutes. A portion of the sperm suspension was diluted, mixed 1:1 with trypan blue, and live sperm were counted on a hemacytometer.

### 3D conformation of the BTB domain

the structure of BTB was retrieved from the Entrez's 3D-structure database http://www.ncbi.nlm.nih.gov/Structure/Entrez/mmdb.shtml, and visualized using the Cn3D application http://www.ncbi.nlm.nih.gov/Structure/CN3D/cn3d.shtml

### Western blot analysis of PLZF

Tissues were disrupted in the Tissue Protein Extraction Reagent (T-per; PIERCE), centrifuged at 4°C for 10 minutes at 10,000 × G, and the supernatant was retained and stored at -80°. Protein concentrations were determined by the BCA method (PIERCE), using a Nanodrop (NanoDrop Technologies, LLC) spectrophotometer. 15 μg protein was mixed with loading buffer containing β-mercaptoethanol, boiled 3 min., and electrophoresced on 10% PAGE gels. Proteins were electrotransferred onto Immobilon-P PVDF membranes (Millipore). The membrane were blocked 1 h at RT in 1×TBS containing 5% skim milk and 0.05% Tween-20. Blots were probed at 4°C overnight in blocking solution with mouse anti-PLZF monoclonal D-9 (Santa Cruz Biotechnology, Inc; 1:1,000 dilution). The blots were then probed with an anti-mouse secondary antibody conjugated to horseradish peroxidase at a 1:3,000 dilution in blocking solution. Blots were washed three times for 5 min after the primary and the secondarychemiluminescence (ECL; PIERCE). Blots were stripped at room temperature in 0.1 M glycine-HCl (pH = 2.5-3.0) for 30 min, before reprobing with 1:10,000 anti β-actin (AC-15, Abcam) mouse monoclonal antibody and goat anti-mouse secondary antibody conjugated to horseradish peroxidase at 1:3,000 dilutions in blocking solution. Blots were washed three times for 5 min after the primary and the secondary antibody probing, and then visualized by chemiluminescence (ECL; PIERCE). Blots were stripped at room temperature in 0.1 M glycine-HCl (pH = 2.5-3.0) for 30 min, before reprobing with 1:10,000 anti β-actin (AC-15, Abcam) mouse monoclonal antibody and goat anti-mouse secondary antibody conjugated to horseradish peroxidase.

## Authors' contributions

YC positionally cloned the 7T mutation, did the axial skeleton analyses and the testis/fertility analyses. She also participated in the writing of the manuscript. LW discovered the mutation, determined its genetic location, and performed initial analyses of the digit defects. JS oversaw the research project from its inception and participated in writing the manuscript. All authors read and approved the final manuscript.

## Supplementary Material

Additional file 1 **Candidate genes in the *7t *critical region**. This file contains a list of t Click here for file

## References

[B1] Costoya JA Pandolfi PP The role of promyelocytic leukemia zinc finger and promyelocytic leukemia in leukemogenesis and development Curr Opin Hematol 2001 8 212 7 10.1097/00062752-200107000-00006 11561158

[B2] Barna M Hawe N Niswander L Pandolfi PP Plzf regulates limb and axial skeletal patterning Nat Genet 2000 25 166 72 10.1038/76014 10835630

[B3] Buaas FW Kirsh AL Sharma M McLean DJ Morris JL Griswold MD de Rooij DG Braun RE Plzf is required in adult male germ cells for stem cell self-renewal Nat Genet 2004 36 647 52 10.1038/ng1366 15156142

[B4] Green MC Luxoid--A new hereditary legand foot abnormality: In the House Mouse J Hered 1955 46 91 99

[B5] Forsthoefel PF The skeletal effects of the luxoid gene in the mouse, including its interactions withthe luxate gene J Morphol 1958 102 247 287 10.1002/jmor.1051020203 13894155

[B6] Costoya JA Hobbs RM Barna M Cattoretti G Manova K Sukhwani M Orwig KE Wolgemuth DJ Pandolfi PP Essential role of Plzf in maintenance of spermatogonial stem cells Nat Genet 2004 36 653 9 10.1038/ng1367 15156143

[B7] Yeyati PL Shaknovich R Boterashvili S Li J Ball HJ Waxman S Nason-Burchenal K Dmitrovsky E Zelent A Licht JD Leukemia translocation protein PLZF inhibits cell growth and expression of cyclin A Oncogene 1999 18 925 34 10.1038/sj.onc.1202375 10023668

[B8] Bardwell VJ Treisman R The POZ domain: a conserved protein-protein interaction motif Genes Dev 1994 8 1664 77 10.1101/gad.8.14.1664 7958847

[B9] Lintermann KG Roth GE King-Jones K Korge G Lehmann M Comparison of the GAGA factor genes of Drosophila melanogaster and Drosophila virilis reveals high conservation of GAGA factor structure beyond the BTB/POZ and DNA-binding domains Dev Genes Evol 1998 208 447 56 10.1007/s004270050202 9799425

[B10] Katsani KR Hajibagheri MA Verrijzer CP Co-operative DNA binding by GAGA transcription factor requires the conserved BTB/POZ domain and reorganizes promoter topology Embo J 1999 18 698 708 10.1093/emboj/18.3.698 9927429 PMC1171162

[B11] Melnick A Carlile G Ahmad KF Kiang CL Corcoran C Bardwell V Prive GG Licht JD Critical residues within the BTB domain of PLZF and Bcl-6 modulate interaction with corepressors Mol Cell Biol 2002 22 1804 18 10.1128/MCB.22.6.1804-1818.2002 11865059 PMC135611

[B12] Barna M Merghoub T Costoya JA Ruggero D Branford M Bergia A Samori B Pandolfi PP Plzf mediates transcriptional repression of HoxD gene expression through chromatin remodeling Dev Cell 2002 3 499 510 10.1016/S1534-5807(02)00289-7 12408802

[B13] Filipponi D Hobbs RM Ottolenghi S Rossi P Jannini EA Pandolfi PP Dolci S Repression of kit expression by Plzf in germ cells Mol Cell Biol 2007 27 6770 6781 10.1128/MCB.00479-07 17664282 PMC2099235

[B14] Sorrentino V Giorgi M Geremia R Besmer P Rossi P Expression of the c-kit proto-oncogene in the murine male germ cells Oncogene 1991 6 149 51 1704118

[B15] Wilson L Ching YH Farias M Hartford SA Howell G Shao H Bucan M Schimenti JC Random mutagenesis of proximal mouse chromosome 5 uncovers predominantly embryonic lethal mutations Genome Res 2005 15 1095 105 10.1101/gr.3826505 16024820 PMC1182222

[B16] Ahmad KF Engel CK Prive GG Crystal structure of the BTB domain from PLZF Proc Natl Acad Sci USA 1998 95 12123 8 10.1073/pnas.95.21.12123 9770450 PMC22795

[B17] Fischer S Kohlhase J Bohm D Heitmann M Schweiger B Hoffmann D Horsthemke B Wieczorek D Biallelic Loss of Function of the Promyelocytic Leukemia Zinc Finger (PLZF) Gene Causes Severe Skeletal Defects and Genital Hypoplasia J Med Genet 2008 45 731 737 10.1136/jmg.2008.059451 18611983

[B18] Blanc I Bach A Lallemand Y Perrin-Schmitt F Guenet JL Robert B A new mouse limb mutation identifies a Twist allele that requires interacting loci on chromosome 4 for its phenotypic expression Mamm Genome 2003 14 797 804 10.1007/s00335-003-2284-x 14724733

[B19] Qu S Niswender KD Ji Q Meer R van der Keeney D Magnuson MA Wisdom R Polydactyly and ectopic ZPA formation in Alx-4 mutant mice Development 1997 124 3999 4008 9374397 10.1242/dev.124.20.3999

