# Supplementary material for: An allele separating skeletal patterning and spermatogonial renewal functions of PLZF
Source: BMC Dev Biol. 2010 Mar 25;10:33. doi: 10.1186/1471-213X-10-33 (PMC2859375; doi:10.1186/1471-213X-10-33)
Supplement: Additional file 1 — Candidate genes in the 7t critical region. This file contains a list of t [file 1471-213X-10-33-S1.DOC]

**Additional File 1**

**Candidate genes in the *7t* critical region**

| **Symbol** | **Name** | **Mouse Phenotypes** | **Mutational analysis** |
| --- | --- | --- | --- |
| *Rexo2* | small fragment nuclease | N/A | dHPLC & Sequencing |
| *Rbm7* | RNA binding motif protein 7 | N/A | Sequencing |
| *Nnmt* | nicotinamide N-methyltransferase | N/A | Sequencing |
| *Zbtb16* | zinc finger and BTB domain containing 16 | limbs/digits/tail/ skeleton/ endocrine/exocrine glands/ reproductive system | dHPLC & Sequencing |
| *Htr3a* | 5-hydroxytryptamine (serotonin) receptor 3A | Life span/aging, endocrine/exocrine, growth/size, hematopoietic, homeostasis,  immune, renal/urinary, reproductive, nervous system/ behavior, touch/vibrissae | Not analyzed |
| *Htr3b* | 5-hydroxytryptamine (serotonin) receptor 3B | N/A | Not analyzed |
| *Usp28* | ubiquitin specific protease 28 | N/A | dHPLC & Sequencing |
| *Zw10* | centromere/kinetochore protein zw10 | N/A | Not analyzed |
